# Leucine-Rich Immune Factor APL1 Is Associated With Specific Modulation of Enteric Microbiome Taxa in the Asian Malaria Mosquito *Anopheles stephensi*

**DOI:** 10.3389/fmicb.2020.00306

**Published:** 2020-02-26

**Authors:** Christian Mitri, Emmanuel Bischoff, Eugeni Belda Cuesta, Stevenn Volant, Amine Ghozlane, Karin Eiglmeier, Inge Holm, Constentin Dieme, Emma Brito-Fravallo, Wamdaogo M. Guelbeogo, N’Fale Sagnon, Michelle M. Riehle, Kenneth D. Vernick

**Affiliations:** ^1^Unit of Insect Vector Genetics and Genomics, Department of Parasites and Insect Vectors, Institut Pasteur, Paris, France; ^2^CNRS Unit of Evolutionary Genomics, Modeling, and Health (UMR2000), Institut Pasteur, Paris, France; ^3^Integromics Unit, Institute of Cardiometabolism and Nutrition, Assistance Publique Hôpitaux de Paris, Pitié-Salpêtrière Hospital, Paris, France; ^4^Bioinformatics and Biostatistics Hub, Department of Computational Biology, Institut Pasteur, Paris, France; ^5^CNRS USR 3756, Institut Pasteur, Paris, France; ^6^Centre National de Recherche et de Formation sur le Paludisme, Ouagadougou, Burkina Faso; ^7^Department of Microbiology and Immunology, Medical College of Wisconsin, Milwaukee, WI, United States

**Keywords:** mosquito, insect immunity, microbiome, leucine-rich repeat, commensalism

## Abstract

The commensal gut microbiome is contained by the enteric epithelial barrier, but little is known about the degree of specificity of host immune barrier interactions for particular bacterial taxa. Here, we show that depletion of leucine-rich repeat immune factor APL1 in the Asian malaria mosquito *Anopheles stephensi* is associated with higher midgut abundance of just the family *Enterobacteraceae*, and not generalized dysbiosis of the microbiome. The effect is explained by the response of a narrow clade containing two main taxa related to *Klebsiella* and *Cedecea*. Analysis of field samples indicate that these two taxa are recurrent members of the wild *Anopheles* microbiome. Triangulation using sequence and functional data incriminated relatives of *C. neteri* and *Cedecea* NFIX57 as candidates for the *Cedecea* component, and *K. michiganensis*, *K. oxytoca*, and *K.sp.* LTGPAF-6F as candidates for the *Klebsiella* component. APL1 presence is associated with host ability to specifically constrain the abundance of a narrow microbiome clade of the *Enterobacteraceae*, and the immune factor may promote homeostasis of this clade in the enteric microbiome for host benefit.

## Importance

Bacteria inhabit the animal digestive tract and body and are generally required for health of the organism. However, some of the bacteria could be harmful if they proliferate to a high level in the host. The mechanisms that allow the host to harbor, control and benefit from bacteria are not well understood. Here we show that a small group of bacteria that are widespread in *Anopheles* mosquitoes appear to be maintained at an appropriate level by the presence of an immune factor, APL1, and that loss of APL1 allows growth of only these few bacterial species.

## Introduction

The animal enteric microbiome is acquired from the environment but is distinct from surrounding free-living environmental microbial communities. A stable commensal consortium is thought to protect the enteric niche from colonization by invading pathogens and carries other potential mutualistic benefits (Buffie et al., 2015; Pamer, 2016). The process of commensalization entails genetic adaptation of the microbe to the physiological conditions of the enteric niche. However, a microbe that adapts so well that it acquires resources too efficiently from the host can be equivalent to a pathogenic infection. Thus, host immunity is required to maintain control over potentially pathogenic microbes adapted to the gut habitat.

Little is known about the microbial specificity, if any, of particular host immune mechanisms that tame otherwise pathogenic free-living microbes and maintain them as commensals (Foster et al., 2017). The same host immune factors that likely evolved to protect against microbial pathogens may also underlie protective tolerance or tethering of certain bacteria as stable commensals in the microbiome (Rakoff-Nahoum et al., 2004; Peterson et al., 2007; Ha et al., 2009; Paredes et al., 2011; Kubinak et al., 2015). The enteric microbiota usually does not trigger an inflammatory response, but rather is probably maintained in a commensal state by a continuous dialogue between host immunity and bacterial microbe-associated molecular patterns and virulence factors. This dialogue in principle leads to homeostasis or resilience of the gut microbiota, based on an equilibrium of benefit and cost for host metabolic activities (Lozupone et al., 2012; Broderick et al., 2014).

In mammals, considerable work has described immunomodulatory, developmental and other effects of the microbiome upon the host (Chung et al., 2012; Gensollen et al., 2016; Honda and Littman, 2016; Thaiss et al., 2016). The influence of the microbiome on a range of host disease states has been described, and microbiome-wide differential abundance analysis is being used for incrimination of specific microbiome taxa underlying disease or health (Carvalho et al., 2012; Kostic et al., 2015; Gilbert et al., 2016; Surana and Kasper, 2017). Relatively fewer studies have examined influence of the host and immunity upon the microbiome. Relevant to the current work, host genetic variation was used to identify immune factors such as TLR5 (Vijay-Kumar et al., 2010) and IgA (Suzuki et al., 2004; Peterson et al., 2007), for which mutants can lead to a dysbiotic state of the enteric microbiome. It was proposed that adaptive immunity “evolved in part to recognize and manage complex communities of beneficial microbes living on or in vertebrates” (McFall-Ngai, 2007). The current work presents evidence suggesting that invertebrate innate immunity may also in part specifically constrain the abundance of certain bacterial taxa, presumably as commensals for host benefit.

In the fruit fly *Drosophila melanogaster*, the closest model organism to mosquitoes, the immune deficiency (Imd) pathway and its negative regulators Caudal and Pirk are mainly responsible for shaping the response to commensal microbiota and maintaining gut homeostasis while simultaneously avoiding an overactive immune response (Ryu et al., 2004; Lhocine et al., 2008). Caudal activity appears to exclude the digestive tube from expression of antimicrobial peptide (AMP) genes, thus protecting commensal microbes, while loss of caudal function allows AMP expression and provokes generalized dysbiosis, including expansion of a *Gluconobacter* taxon, leading to host death (Ryu et al., 2008). In *Bactrocera dorsalis*, a fruit fly native to Southeast Asia, depletion of the dual oxidase gene *BdDuox* caused global shifts of the enteric flora characterized by elevated overall bacterial density, higher species richness probably due to expansion of rare taxa, and the decrease of *Enterobacteriaceae* and *Leuconostocaceae* (Yao et al., 2016). Investigation of *D. melanogaster* host immune interaction with the gut microbiome has described the spatial ecology of microbial distribution along the digestive tube, the basal immune activity that appears to provide a hospitable environment for different taxa, and the effect of the microbiome on intestinal stem cell proliferation (Buchon et al., 2013). Genetic analysis of *D. melanogaster* under altered nutritional regimes revealed genes associated with microbial variation (Dobson et al., 2015). However, except for caudal, specific interactions of insect host immune factors with particular microbial taxa have not been described.

The mosquito microbiome can influence mosquito infection and transmission of pathogens such as malaria parasites and arboviruses, with contrasting effects depending on pathogen and vector species. For *Plasmodium*, the mosquito gut microbiota has most often been described as antagonistic to infection (Pumpuni et al., 1993, 1996; Oduol et al., 2000; Frolet et al., 2006; Dong et al., 2009; Rodrigues et al., 2010; Cirimotich et al., 2011), although in wild mosquitoes carriage of *Enterobacteriaceae* may positively correlate with *Plasmodium* susceptibility (Boissiere et al., 2012). The microbiome can have contrasting effects on arbovirus infection. The presence of the live gut microbiome was required for full infectivity of the alphavirus O’nyong-nyong to *Anopheles* (Carissimo et al., 2015), while the enteric flora was antagonistic for infection of the flavivirus dengue in *Aedes* (Xi et al., 2008), although neither of these studies identified specific microbiome taxa associated with the phenotypes. The mechanisms by which the enteric flora influence mosquito susceptibility to pathogens are not known, but may involve stimulation of basal signaling by immune pathways (Frolet et al., 2006; Dong et al., 2009; Blumberg et al., 2013). The exposure of the epithelium to gut microbiota during *Plasmodium* infection also triggers immune hemocyte differentiation (Rodrigues et al., 2010). Finally, elements of the microbiome may influence insecticide resistance phenotypes in mosquitoes (Dada et al., 2018). Thus, microbiome effects on mosquito vector biology may be multiple and important, but are not yet well understood.

In mosquitoes, the host factors that structure enteric microbiome communities remain little known. Evidence indicates that bacterial recognition is more fine-grained than the simple Gram-positive and Gram-negative dichotomy. It was found that *Anopheles* host genetic variation influenced the response to exogenous *Serratia marcescens*, and silencing of candidate genes encoding type III fibronectin domain proteins caused shifts in *Serratia* abundance, but specific taxa were not profiled (Stathopoulos et al., 2014). The *Anopheles* ortholog of Down syndrome cell adhesion molecule gene, AgDscam, displays differential transcript splicing and protein binding to several bacteria tested, although a role as an enteric barrier factor policing the microbiome is not known (Dong et al., 2006). Bacteria of the genus *Asaia* are found in the enteric microbiome of wild *Anopheles* and confer host benefit, but host mechanisms of their maintenance as commensals are not known (Chouaia et al., 2012; Rami et al., 2018). In *Aedes* mosquitoes, C-type lectins coat the surface of enteric bacteria and protect them from attack by antimicrobial peptides (Pang et al., 2016). However, despite the apparent bacterial preference seen in these mosquito interactions, fine taxonomic specificity has not yet been demonstrated.

The current work presents evidence suggesting specific modulation of a natural enteric microbiome clade by the *Anopheles stephensi* immune factor, APL1. This protein was previously known from studies of the African malaria vectors of the Gambiae species complex, *A. gambiae* and *A. coluzzii*, to limit malaria parasite infection of the midgut epithelium after an infectious bloodmeal (Riehle et al., 2006; Fraiture et al., 2009; Povelones et al., 2009; Mitri et al., 2015). APL1 is expressed and secreted from hemocytes into the systemic hemocoel compartment, and was not previously reported to influence bacterial levels or mosquito mortality. It was recently found that APL1 is present as a single ancestral gene in most *Anopheles* including *A. stephensi*, but has expanded to three paralogs in an African lineage that includes the Gambiae species complex (Mitri et al., 2019). Silencing of the unique APL1 copy in *A. stephensi* resulted in significant mosquito mortality that was rescued by antibiotic treatment, suggesting that changes in the enteric microbiome underlie the phenotype (Mitri et al., 2019). However, it was not known whether the altered microbiome in the absence of APL1 was due to changes of specific taxa, or generalized dysbiosis of the microbiome. Here, we profile the taxonomic structure of the *A. stephensi* enteric microbiome in the presence and absence of APL1 and antibiotic treatment. The combination of 16S rRNA amplicon sequence, microbiome differential abundance, shotgun metagenomic sequencing of the enteric microbiome, and field studies identified just two bacterial candidate taxa, widespread members of the wild *Anopheles* microbiome, that expand in abundance after depletion of APL1.

## Results

### Metagenomic Identification of Bacterial Taxa Modulated by Presence of APL1

We taxonomically profiled the bacteria in *A. stephensi* midguts in the presence and absence of APL1 and antibiotics, and analyzed differential bacterial abundance. The V4 hypervariable region of the 16S ribosomal RNA (rRNA) gene was amplified and sequenced from DNA of *A. stephensi* midguts dissected from mosquitoes treated with dsAPL1, dsGFP, antibiotics, or no antibiotics, each in three biological replicates (sample list, Supplementary Table S1).

Bacterial operational taxonomic units (OTUs) were picked, taxonomically assigned, and counted using the SHAMAN workflow (Quereda et al., 2016), which incorporates the program DESeq2 (Love et al., 2014) for analysis of quantitative metagenomics data and differential abundance using a multi-factor generalized linear model. A total of 457 OTUs were identified in midguts among all treatment conditions combined (clustered OTU sequences, Supplementary File S1). In all, 279 (61%) of these OTUs were annotated in the SILVA rRNA taxonomic database and were provisionally named on that basis (annotated raw OTU matrix, Supplementary Table S2). OTU counts were normalized (normalized OTU matrix, Supplementary Table S3) and analyzed to detect differentially abundant taxa found in two different comparisons: (i) mosquitoes depleted for APL1 versus controls with APL1, both without antibiotic treatment (OTU significance, Supplementary Table S4); and (ii) mosquitoes treated with antibiotics versus controls without antibiotics, both with APL1 depleted (OTU significance, Supplementary Table S5).

**FIGURE 1:**
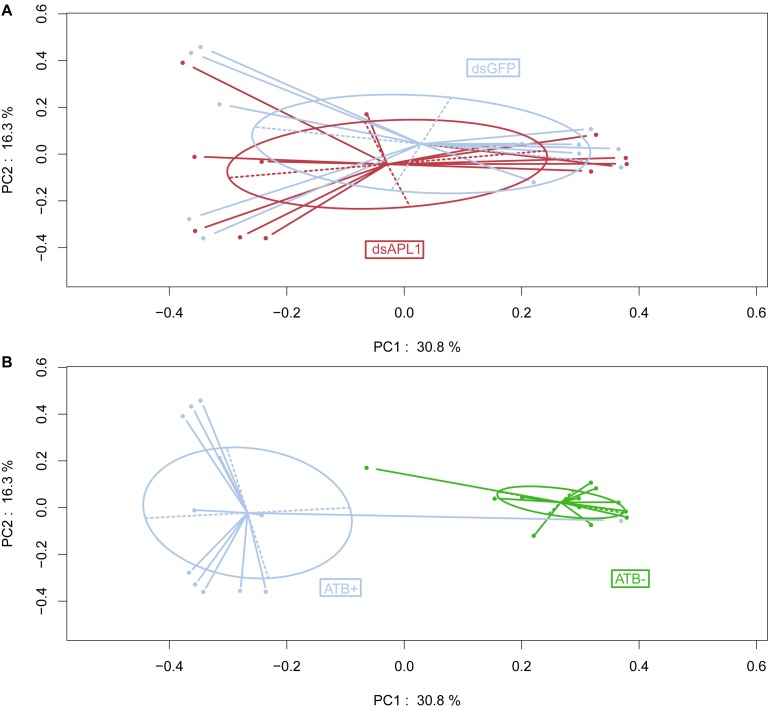
Influence of APL1 or antibiotics on bacterial taxa in ***Anopheles stephensi*.** Principal Coordinates Analysis (PCoA) of OTU distributions between mosquito treatments. **(A)** Mosquitoes depleted for APL1 as compared to controls. APL1 was depleted by treatment with dsAPL1 (dsAPL1), or controls were treated with dsGFP (dsGFP). Permutational multivariate analysis of variance (PERMANOVA) *p* = 0.696 indicates absence of significant global effect of treatments upon OTU distributions. **(B)** Mosquitoes treated with antibiotics as compared to controls. Mosquitoes were treated with antibiotics (ATB+) or without antibiotics (ATB-). PERMANOVA *p* = 0.001 indicates significant effect of treatments upon OTU distributions.

Examination of the data by Principal Coordinates Analysis (PCoA) indicated that overall OTU abundance distributions were not significantly different in the mosquitoes depleted for APL1 as compared to controls (Figure 1A, *p* = 0.696). This suggests that the depletion of APL1 was not associated with a global taxonomic shift but was likely restricted to specific taxa. In marked contrast, mosquitoes treated with antibiotics displayed radically separated patterns of OTU abundance as compared to untreated mosquitoes (Figure 1B, *p* = 0.001), which is consistent with the expectation of widespread shifts due to suppression of antibiotic sensitive taxa. Finally, examination of biological replicates indicates that distributions of replicates 1 and 3 for all treatments combined are largely superimposed, while the replicate 2 distribution is partly overlapping with the other two (Supplementary Figure S1, *p* = 0.019). The replicates display relatively minor levels of differentiation considering that replicates were generated from distinct oviposition events collected from non-overlapping generations of an *A. stephensi* colony.

The frequency distribution of mean normalized counts of all OTUs across all treatment conditions reveals an inflection at the value of 1903 counts (Supplementary Figure S2). We employ this natural gap as the threshold distinguishing eight major OTUs comprising the *A. stephensi* core enteric microbiome, which encompassed 93% of total bacterial abundance (the remaining 7% of abundance to the left of the inflection was comprised of 449 OTUs).

**FIGURE 2:**
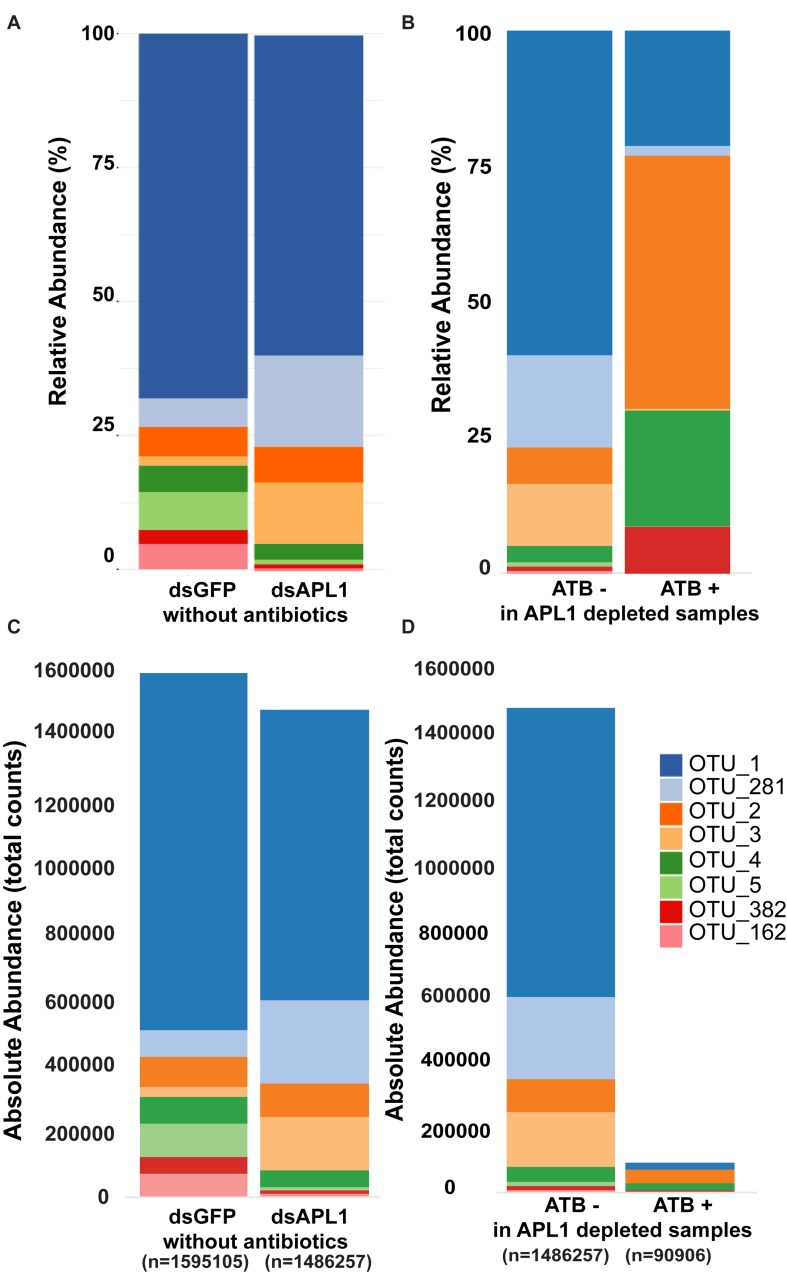
Representation of OTUs after APL1 depletion or antibiotic treatment in *Anopheles stephensi*. The representation of the eight major OTUs in *A. stephensi* comprising 93% of enteric bacterial abundance is shown as relative abundance **(A,B)** or absolute abundance **(C,D)**. **(A)** Relative abundance of the OTUs after APL1 depletion by treatment with specific double-stranded RNA (dsAPL1) as compared to controls (dsGFP), all mosquitoes without antibiotics (data in Supplementary Table S4). **(B)** Relative abundance of OTUs after antibiotic treatment (ATB+) as compared to no antibiotics (ATB-), all mosquitoes depleted for APL1 (data in Supplementary Table S5). **(C)** Absolute abundance of OTUs after APL1 depletion without antibiotics (the same data as in panel **A**), or **(D)** Absolute abundance of OTUs after antibiotic treatment with APL1 depleted (the same data as in panel **B**).

The core enteric microbiome of eight taxa (Supplementary Table S3) is comprised of OTU1_Serratia (58% of total bacteria), OTU382_Serratia (2.5%), OTU162_Serratia (2.2%), OTU 281_Cedecea (9.3%), OTU2_Elizabethkingia (8.5%), OTU3_Klebsiella (5.4%), OTU4_Asaia (5.6%), and OTU5_Aeromonas (3.4%). Qualitative examination of relative abundance of the eight major OTUs suggested that depletion of APL1 led to altered representation among these major OTUs (Figure 2A and Supplementary Table S4). Treatment with antibiotics provoked a generalized alteration of OTU relative abundance (Figure 2B). Examination of absolute rather than relative abundance of OTUs indicates that the total bacterial loads are comparable in mosquitoes with and without APL1 (Figure 2C). The samples without antibiotics had ∼1.5–1.6 M total normalized sequence reads mapping to the eight major OTUs (Figure 2C, dsGFP and dsAPL1). In contrast, antibiotic treatment decreased the overall bacterial absolute abundance levels by approximately two orders of magnitude (Figure 2D, compare ATB- and ATB). Consequently, the relative abundance of OTUs in the antibiotic treated samples (ATB+ in Figure 2B) represents relative proportions of the eight major taxa in a drastically reduced total number of absolute OTU counts, and thus is not comparable to the relative OTU proportions without antibiotic (ATB- in Figure 2B).

**FIGURE 3:**
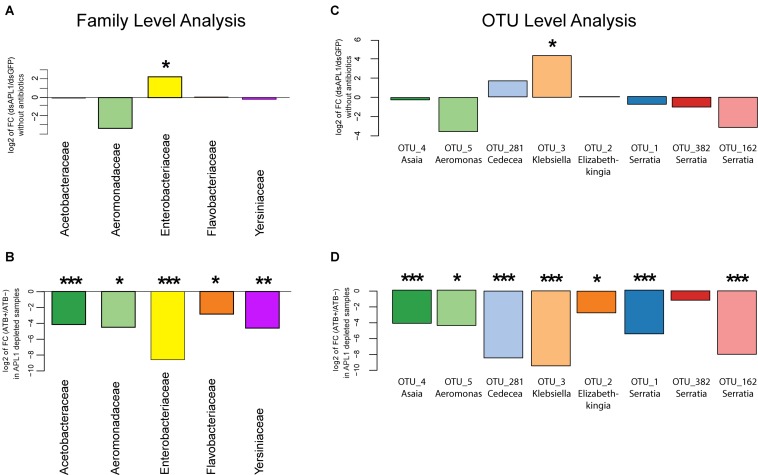
Absolute abundance change of bacterial taxa after APL1 depletion or antibiotic treatment in *Anopheles stephensi*. The change in absolute abundance of bacterial families **(A,B)** or the eight major OTUs **(C,D)** in panel **(A)**. stephensi comprising 93% of enteric bacterial abundance is shown as the log2 value of fold change between indicated conditions. Absolute normalized bacterial abundance values were tested for departure from the null hypothesis of zero fold change, with nominal *p*-values adjusted by the Benjamini and Hochberg procedure (see section “Materials and Methods”). **(A)** Fold-change of absolute abundance of bacterial families after APL1 depletion by treatment with specific double-stranded RNA (dsAPL1) as compared to controls (dsGFP), all mosquitoes without antibiotics (data in Supplementary Table S4). Positive values indicate increased absolute abundance of the family in APL1 depleted samples and negative values indicate decreased abundance after APL1 depletion. **(B)** Fold-change of absolute abundance of bacterial families after antibiotic treatment (ATB+) as compared to no antibiotics (ATB-), all mosquitoes depleted for APL1 (data in Supplementary Table S5). Negative values indicate decreased absolute abundance of the family after antibiotic treatment. **(C)** Fold-change of absolute abundance of the eight major OTUs after APL1 depletion by treatment with specific double-stranded RNA (dsAPL1) as compared to controls (dsGFP), all mosquitoes without antibiotics (data in Supplementary Table S4). Positive values indicate increased absolute abundance of the OTU in APL1 depleted samples and negative values indicate decreased abundance after APL1 depletion. **(D)** Fold-change of absolute abundance of the 8 major OTUs after antibiotic treatment (ATB+) as compared to no antibiotics (ATB-), all mosquitoes depleted for APL1 (data in Supplementary Table S5). Negative values indicate decreased absolute abundance of the OTU after antibiotic treatment. Adjusted significance levels, *p-value < 0.05, **p-value < 0.01, and ***p-value < 0.001. All values for fold-change, log2 fold-change and *p*-value for APL1 depletion and antibiotic treatment are in Supplementary Tables S4, S5, respectively.

Next, we statistically analyzed the effect of APL1 depletion and antibiotic treatment upon absolute normalized bacterial abundance of the *A. stephensi* core enteric microbiome, totaling 93% of total bacterial abundance. We tested for departure from the null hypothesis that fold-change for a given bacterial family or OTU is zero, with nominal *p*-values adjusted by the Benjamini and Hochberg procedure (see section “Materials and Methods”). Analyzed at the taxonomic family level, only the *Enterobacteriaceae* displayed significantly altered absolute normalized abundance after APL1 depletion (Figure 3A, 4.9-fold increased absolute normalized abundance after APL1 depletion, *p* = 0.02, Supplementary Table S4). All of the families of the core enteric microbiome were significantly antibiotic sensitive, including the APL1-responsive *Enterobacteriaceae* (Figure 3B and Supplementary Table S5). Analyzed at the OTU level, the APL1-sensitive *Enterobacteriaceae* family included two members, OTU3_Klebsiella and OTU281_Cedecea (Figure 3C). Of these, OTU3_Klebsiella displayed significantly increased absolute normalized abundance after depletion of APL1 as compared to dsGFP treated controls (18-fold increased absolute abundance, *p* = 0.012, Supplementary Table S4) while OTU281_Cedecea displayed a non-significant tendency of increased absolute abundance after APL1 depletion (3.3-fold increased absolute abundance, *p* = 0.219). Because the non-significant OTU281_Cedecea is in the same family as the significant OTU3_Klebsiella and displays a tendency in the same direction for APL1-depleted and antibiotic treated mosquitoes, it is a taxon of potential interest and it is hereafter analyzed jointly with OTU3_Klebsiella. Nevertheless, inclusion of OTU281_Cedecea should be regarded as provisional, and further studies will be necessary to determine the actual level of OTU281_Cedecea sensitivity to APL1 activity.

**FIGURE 4:**
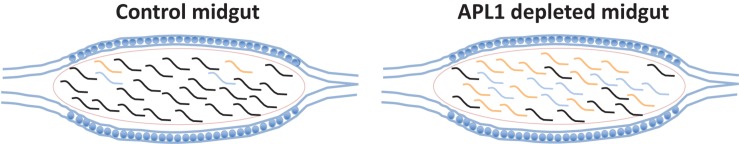
Differential abundance of bacterial taxa associated with presence of APL1. Diagram summarizes bacterial abundance within the midguts of mosquitoes in presence or absence of APL1. Curved lines indicate the different bacterial clades present in the mosquito midgut. The two bacterial taxa of the APL1-responsive *Enterobacteriaceae* are indicated in color (OTU3_Klebsiella, orange and OTU281_Cedecea, blue), while other taxa with abundance not associated with APL1 are indicated by black lines. In mosquitoes with the APL1 gene silenced and in the absence of antibiotics (**right column**, APL1 depleted) the clade of two *Enterobacteriaceae* OTUs expands in relative and absolute abundance, but without altering the total bacterial load as compared to control mosquitoes, also in the absence of antibiotics (**left column**, Control). The two *Enterobacteriaceae* taxa, OTU3_Klebsiella and OTU281_Cedecea, are sensitive to antibiotics (Figure 3D). The condition that produces significant host mortality in *A. stephensi* (Mitri et al., 2019) is illustrated by the midgut on the right.

All but one (OTU382_Serratia) of the eight major OTUs were significantly antibiotic sensitive under APL1-silenced conditions (Figure 3D and Supplementary Table S5). Notably, OTU3_Klebsiella and OTU281_Cedecea are the only major OTUs that expanded (or displayed tendency) in absolute abundance after APL1 depletion, and were also antibiotic sensitive. Two other taxa displayed a non-significant tendency of decreased abundance after APL1 depletion (OTU5_Aeromonas and OTU162_Serratia), but these two were then further decreased by antibiotic treatment. Because these two taxa respond in the same direction to both depletion of APL1 and treatment with antibiotics, their response pattern could not underlie the observed mortality phenotype associated with APL1 depletion that was complemented by antibiotics (Mitri et al., 2019). Thus, the results are most consistent with the interpretation that APL1 activity is associated with specific homeostasis of OTUs of the *Enterobacteriaceae* family within the enteric microbiome, OTU3_Klebsiella and possibly OTU281_Cedecea, which expand in absolute and relative abundance after APL1 depletion. The differential abundance effects upon bacterial taxa associated with presence of APL1 are summarized in Figure 4.

### Phylogenetic Placement and Molecular Confirmation of OTUs Associated With APL1

For phylogenetic placement, consensus sequences of the OTU3_Klebsiella and OTU281_Cedecea 16S V4 Illumina amplicons were clustered in a reference 16S rRNA gene phylogenetic tree of 2807 full-length 16S rRNA gene sequences from complete prokaryotic genomes in the GenBank database (Supplementary Figure S3, OTUs are indicated in the tree by blue highlight and arrows). OTU281_Cedecea groups with *Cedecea*, in a basal position of the *Enterobacteriaceae* clade comprised of the taxa *Escherichia/Shigella, Salmonella, Enterobacter* and *Klebsiella*. Concordant with this placement, the OTU281_Cedecea 16S V4 Illumina amplicon displays 100% nucleotide identity with 16S rRNA gene sequences from the complete prokaryotic genomes of *Cedecea lapagei, Cedecea neteri* and *Cedecea davisae*. The *Cedecea* genus comprises rare bacteria of which only several species are known, mainly identified from environmental surveys (Chan and Tan, 2017). The OTU3_Klebsiella taxon groups closer to the *Enterobacter-Klebsiella* clade, displaying 100% nucleotide identity between the 16S V4 Illumina amplicon and *Enterobacter-Klebsiella* 16S rRNA gene sequences. Members of the *Klebsiella* genus are frequent constituents of animal microbiomes. Thus, the two *Anopheles* OTUs sensitive to the presence of APL1 belong to a relatively narrow clade of the *Enterobacteriaceae*.

In order to validate OTU differential abundance after APL1 depletion, we used the above phylogenetic sequence data (Supplementary Figure S3) to design quantitative PCR (qPCR) assays to measure abundance of OTU3_Klebsiella and OTU281_Cedecea. We aligned the 16S V4 Illumina amplicon sequences with nearest phylogenetic relatives from the above 16S tree, and used sequence information extending beyond the 16S V4 Illumina amplicons to design qPCR primers discriminant for the two OTUs (Supplementary Figure S4 and Supplementary Table S6). The resulting qPCR assays use primers distinct from the original 16S V4 Illumina amplicon primers, and thus comprise independent assays putatively recognizing the same two OTU targets. The qPCR product sequences cluster near the V4 amplicon sequences (Supplementary Figure S3, qPCR products are indicated in the tree by purple highlight and arrows).

**FIGURE 5:**
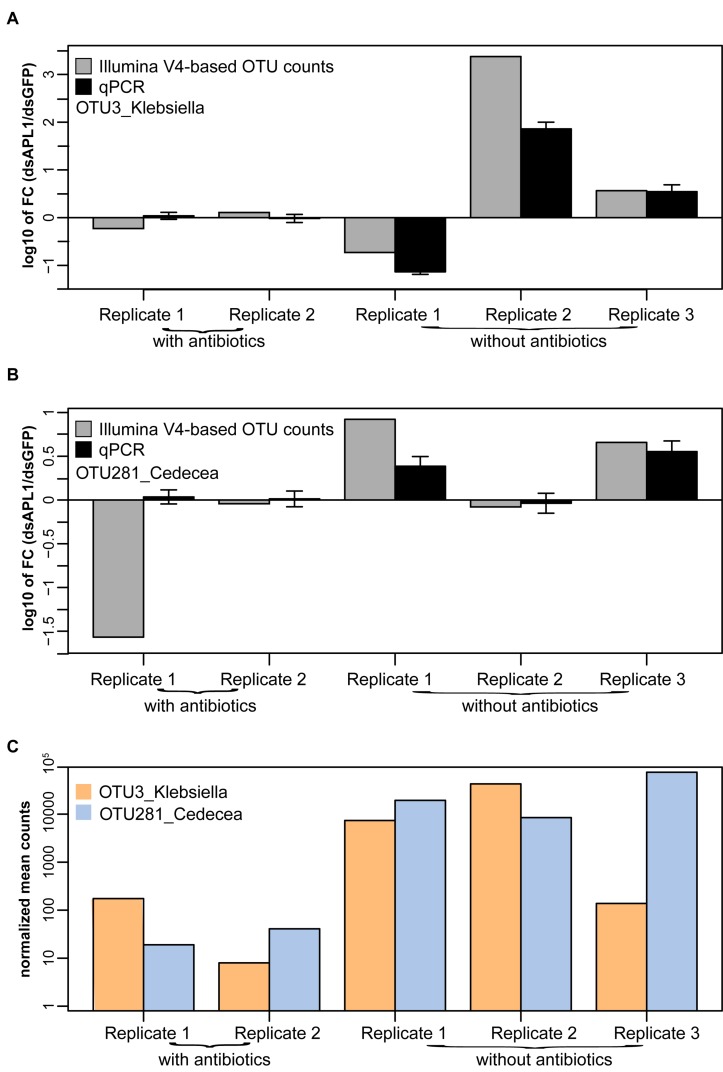
Validation by quantitative PCR of OTUs associated with APL1. Differential absolute abundance of OTUs in *A. stephensi* after APL1 depletion as compared to controls measured by quantitative PCR (qPCR, black bars) and by Illumina metagenomic sequencing of the 16S rRNA gene V4 amplicon (Illumina V4-based OTU counts, gray bars), for panel **(A)** OTU3_Klebsiella and **(B)** OTU281_Cedecea. Bars indicate the ratio of OTU absolute abundance after APL1 depletion as compared to controls. X-axis indicates replicate samples from mosquitoes treated with dsRNA without antibiotics (corresponding to Figure 3C), or the same dsRNAs with antibiotics (corresponding to Figure 3D). Concordance between qPCR and Illumina amplicon sequence values for the two OTUs display correlation coefficient *r*^2^ = 0.69. **(C)** Illumina V4-based normalized mean counts in each sample for OTU3_Klebsiella and OTU281_Cedecea. For qPCR, the *A. stephensi* ribosomal protein rpS7 gene was used as internal calibrator (result was equivalent using instead total 16S rRNA gene DNA as calibrator, Supplementary Figure S5). Error bars indicate 95% confidence interval for qPCR.

The qPCR assays were applied to the mosquito midgut DNA samples analyzed for microbiome differential abundance. Molecular OTU abundance measurements by the qPCR assays were concordant with most of the OTU abundance measurements derived from sequencing of the 16S V4 Illumina amplicons (OTU3_Klebsiella, Figure 5A; OTU281_Cedecea, Figure 5B; correlation coefficient between qPCR and Illumina metagenomic-based OTU counts, *r*^2^ = 0.69). The only discordant comparison was OTU 281_Cedecea in Replicate 1 with antibiotics (Figure 5B), for which there were very few normalized OTU counts detected by 16S V4 amplicon sequencing because of the suppressive effect of the antibiotics (Figure 5C). The qPCR results were consistent with the results from the original metagenomic OTU counts whether target DNA abundance was calibrated against total mosquito DNA or against total bacterial DNA (*r*^2^ = 0.9991 for internal calibrator *A. stephensi* ribosomal protein rpS7 gene or 16S rRNA gene, Supplementary Figure S5). Thus, specific quantification by qPCR confirms the abundance estimates based on metagenomic OTU counts, validates the qPCR assays, and reinforces the altered differential abundance in the microbiome of OTU3_Klebsiella and OTU281_Cedecea associated with the depletion of APL1.

### Shotgun Sequencing and Fine Phylogenetic Placement of OTUs Associated With APL1

To refine the phylogenetic placement of OTU3_Klebsiella and OTU281_Cedecea, we performed shotgun metagenomics on DNA from midguts of APL1-depleted *A. stephensi* mosquitoes. APL1-depleted samples were sequenced because they harbor the highest abundance of the relevant APL1-modulated OTUs (Figures 2, 3). Of 462 million total paired-end 150 bp reads, mosquito reads were removed. From the non-mosquito fraction, the reads for the *Cedecea* and *Klebsiella* clades were binned and taxonomically identified by searching against a database of reference genomes using Centrifuge (Kim et al., 2016). The *Cedecea* and *Klebsiella* fractions included ∼3.2 million reads each, ∼1% of the total sequence generated, which allowed estimation of the species composition and abundance of *Cedecea* and *Klebsiella* taxa (Supplementary Figure S6). The midgut *Cedecea* fraction was dominated at the species level by *C. neteri* (76% of the *Cedecea* midgut shotgun sequences) and *Cedecea* NFIX57 (23%), whereas the *Klebsiella* fraction contained three species with similar abundance profiles, *K. michiganensis* (38% of *Klebsiella* midgut shotgun sequences), *K. oxytoca* (34%), and *K.sp*.LTGPAF-6F (23%).

We combined three lines of evidence to infer the most likely taxonomic identities of the OTUs in the *Enterobacteriaceae* sensitive to the presence of APL1 by aligning (i) the original 16S V4 Illumina amplicons, (ii) the sequences of the qPCR extended amplicons, and (iii) complete 16S rRNA gene sequences from the GenBank assemblies of the above *Cedecea* and *Klebsiella* species identified in the midgut shotgun dataset. The qPCR primers external to the OTU281_Cedecea 16S V4 Illumina amplicon display 100% identity to 16S genes corresponding to 5/5 available *C. neteri* genome assemblies in Genbank (100%), and 1/1 (100%) *Cedecea* NFIX57 genome assemblies (Supplementary Figure S7). Thus, the qPCR assay for OTU281_Cedecea should accurately amplify and measure both of these *Cedecea* species identified in *A. stephensi* midgut by the shotgun sequence data. The former species was the more abundant of the two in the shotgun sequence data. Based on the same criteria, the qPCR primers external to the OTU3_Klebsiella 16S V4 amplicon display 100% identity and should efficiently amplify the 16S gene in 16/61 *K. michiganensis* genomes (26.22%), 1/86 *K. oxytoca* genomes (1.13%), and 1/1 (100%) *K.sp.* LTGPAF-6F genomes (respectively, Supplementary Figures S8–S10).

The above species alignments also reveal that the qPCR assays are each diagnostic for an oligotype (sensu, Eren et al., 2013) distinguished by four fixed nucleotide variants. The *Cedecea* oligotype carries TTAT at the four diagnostic positions and includes *C. neteri* and *Cedecea* NFIX57 (Supplementary Figure S7), while the *Klebsiella* oligotype carries CCGG at the discriminant positions and includes *K. michiganensis*, *K. oxytoca*, and *K.sp.* LTGPAF-6F (Supplementary Figures S8–S10). The components of *Klebsiella* targeted by the qPCR assay appear less well defined taxonomically, or may display greater diversity, than the *Cedecea* group. These three lines of evidence (original 16S V4 Illumina amplicon OTUs, qPCR extended amplicons, and shotgun sequence species) intersect to confirm the microbiome differential abundance and qPCR results, and coherently support the identities of these two OTUs in the differential analysis. The data support an interpretation of each of the two OTUs as closely related taxonomic swarms rather than single taxonomic entities. Metagenomic shotgun assembly was able to narrow the taxonomic space occupied by each OTU, but existing phylogenetic databases are incomplete for these taxa. Finer scale resolution and incrimination of specific taxa will require additional complete genome assemblies to identify the taxa comprising the *Cedecea* and *Klebsiella* oligotypes. Specific diagnostic assays would be needed to facilitate isolation, culture and controlled experiments using individual taxa.

### Candidate *Klebsiella* and *Cedecea* OTUs Are Present in the Wild *Anopheles* Microbiome

**FIGURE 6:**
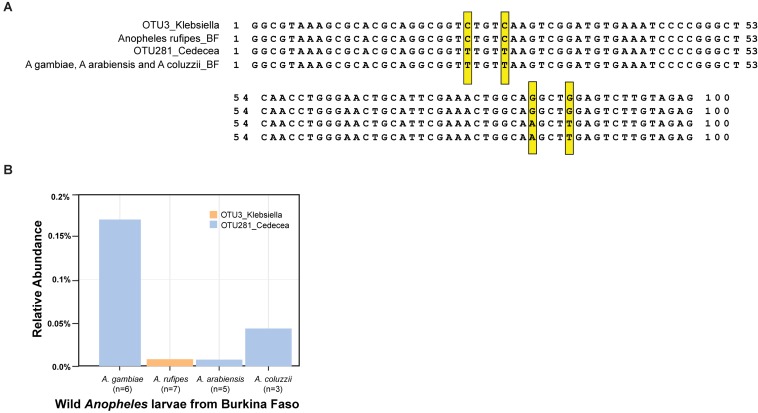
Carriage of OTU3_Klebsiella and OTU281_Cedecea by wild *Anopheles*. DNA was isolated from samples collected from larval pools in Burkina Faso, species typed as *A. gambiae*, *A. coluzzii*, *Anopheles rufipes*, and *A. arabiensis*. Amplicons of the V4 hypervariable region of the 16S rRNA gene were sequenced, and OTUs were identified. **(A)** Sequence alignment displays sequence of OTU3_Klebsiella from the *A. stephensi* laboratory colony (line 1) and consensus sequence from wild *A. rufipes* (*n* = 7, line 2), OTU281_Cedecea from *A. stephensi* (line 3), and consensus sequence from wild *A. gambiae* (*n* = 6), *A. arabiensis* (*n* = 5), and *A. coluzzii* (*n* = 3, line 4). Exact matches to the oligotype diagnostic positions shared across laboratory *A. stephensi* and wild samples are indicated (yellow boxes). Position 1 of the alignment is equivalent to position 569 of the 16S rRNA gene sequence of *Cedecea neteri* strain M006 (NCBI genome reference NZ_CP009458.1, locus_LH23_RS00335). **(B)** The relative abundance of OTU3_Klebsiella and OTU281_Cedecea in the wild microbiome samples. Sample sizes are indicated for the number of mosquito larvae contributing per species pool.

Wild *Anopheles* populations harbor both of the OTUs in the *Enterobacteriaceae*, which is sensitive to the activity of APL1, as defined most precisely to the level of the *Cedecea* and *Klebsiella* oligotypes. Wild *A. stephensi* collected in India (Rani et al., 2009) carried bacteria with an exact sequence match to the OTU3_Klebsiella 16S V4 sequence from the Illumina amplicons and the qPCR product, including the diagnostic CCGG oligotype positions. Wild *Anopheles* larvae sampled from Burkina Faso carried bacteria with an exact match to OTU281_Cedecea 16S V4 Illumina amplicon and qPCR sequence, including the TTAT oligotype positions, and to bacteria matching OTU3_Klebsiella, including CCGG oligotype positions (Figure 6A). In addition to A. stephensi, the two bacterial taxa display consistent presence in the population of multiple African *Anopheles* species sampled, including *A. gambiae*, *A. coluzzii*, *A. arabiensis*, and *A. rufipes* (Figure 6B). Thus, carriage of these two OTUs is not an attribute restricted to insectary reared *Anopheles*, and the OTU3_Klebsiella and OTU281_Cedecea taxa appear to be recurrent commensal members of the *Anopheles* microbiome in geographically widespread populations and host species.

## Discussion

Here we identify an association of the LRR immune factor APL1 with the fine structuring of the enteric bacterial microbiome in the Asian malaria vector *A. stephensi*. APL1 depletion is associated with the expansion of a clade within the *Enterobacteriaceae* comprised of two detected bacterial OTUs, identified as OTU3_Klebsiella, which displayed a significant effect, and OTU281_Cedecea, which displayed a non-significant tendency in the same direction. OTU281_Cedecea was analyzed jointly as a taxon of potential interest given the phylogenetic proximity of the two OTUs, but further studies of the bacterial spectrum of APL1 influence will be necessary to determine the actual level of OTU281_Cedecea sensitivity to APL1 activity.

A combination of 16S rRNA amplicon sequence, microbiome differential abundance, diagnostic qPCR oligotyping, shotgun metagenomic sequencing of the enteric microbiome, and field studies allowed triangulation among known taxa to incriminate relatives of *C. neteri* and *Cedecea* NFIX57 as candidates for OTU281_Cedecea, and *K. michiganensis*, *K. oxytoca*, and *K.sp.* LTGPAF-6F as candidates for OTU3_Klebsiella. These taxa are poorly represented in genome databases. The finest level of resolution developed here for the two OTUs, which is the diagnostic oligotype qPCR assays, likely still detect closely related taxonomic swarms rather than single taxonomic entities. Complete genome assemblies will be needed to resolve the OTUs to single taxa, in order to characterize cultured taxa for controlled studies.

There are likely to be interactions between microbiome taxa in the consortium. This may be the case for the taxa OTU3_Klebsiella and OTU281_Cedecea, based on the observation that biological replicates 1 and 2 carry one or the other OTU, and only replicate 3 carries both of these major OTUs of the *Enterobacteriaceae* (Figure 5). The close phylogenetic placement of the two OTUs would be consistent with occupation of a similar ecological niche in the microbiome, provision of similar host benefits, and possible redundancy of the two taxa in the microbiome consortium. This apparent potential alternation of the OTUs among biological replicates could explain at least in part why OTU281_Cedecea displayed only a non-significant tendency of response to APL1 depletion. Further ecological study of interactions within the microbiome consortium, including as a consequence of the destabilizing effect of APL1 depletion, would be required.

The differential abundance of the *Enterobacteriaceae* in the absence of APL1 is relevant to the previous observation that depletion of APL1 was associated with significantly elevated *A. stephensi* mortality (Mitri et al., 2019). The two *Enterobacteriaceae* OTUs are present in natural *Anopheles* populations including wild *A. stephensi*, indicating they are natural members of the *Anopheles* microbiome. Their widespread presence in the *Anopheles* microbiome suggests they are likely to be commensals conferring unknown fitness benefits to the mosquito host. If true, then some aspect of APL1 function would be associated, at least in part, with the maintenance of this enteric bacterial clade in a commensal state. Particularly interesting are the correlations between depletion of APL1, differential abundance of the narrow *Enterobacteriaceae* clade, and host mortality. Further work will be required to determine the causality, if any, underlying these correlations, and the relevant mechanisms. Three main questions that will require further work are discussed in order below.

First, if APL1 activity is causal for host control of the *Klebsiella-Cedecea* clade of the *Enterobacteriaceae*, what is the mechanism? APL1 has been little studied in *A. stephensi*, but the ortholog APL1C in *A. gambiae* and *A. coluzzii* acts as a subunit of a ternary immune complex including the LRR protein LRIM1, and the complement-like factor TEP1 (Blandin et al., 2004; Riehle et al., 2008; Povelones et al., 2009; Williams et al., 2019). The immune complex is known to protect against *Plasmodium* infection, and an antibacterial function has not been reported for APL1C. Because the presence of the immune complex has only been detected in *A. gambiae* and *A. coluzzii*, it cannot be excluded that APL1 could function very differently in *A. stephensi*. Nevertheless, a recent report indicated that *A. coluzzii* APL1 was processed by a limited C-terminal cleavage specifically after mosquito injection with the *Enterobacteriaceae, Escherichia coli*, but not with the Gram positive *Staphylococcus aureus* (Reyes Ruiz et al., 2019).

The simplest mechanism for APL1 to limit the bacterial OTUs would involve direct recognition and binding of APL1 to the specific bacterial taxa, initiating a targeted effector response. However, given that the OTUs are members of the enteric microbiome, and potentially beneficial commensals, the functional endpoint of APL1 activity toward them would presumably be policing and protective tolerance to tether the OTUs in homeostasis, rather than elimination. Alternately, the APL1 interaction with bacteria could be indirect, involving for example, APL1-dependent stimulation of immune signaling followed by release of bacterial clade-specific antimicrobial peptides (AMPs), but subject to the same caveat that the OTUs are not, in principal, pathogens unless uncontrolled. In addition to APL1, it is likely that other immune-related genes may be involved in specific bacterial interactions that structure the microbiome, which will require further work to determine.

Second, what is the host physiological site where the *Klebsiella-Cedecea* clade is surveyed and limited within the mosquito? The barrier could be established by APL1 within the midgut lumen, although there is no evidence in the better-studied *A. gambiae* and *A. coluzzii* mosquitoes for APL1C expression by the midgut epithelium, or presence of the protein in the lumen. In *Aedes* mosquitoes, C-type lectins are localized within the midgut lumen, and binding to bacteria protects them from clearance by AMPs (Pang et al., 2016). Alternately, the barrier could occur in the hemocoel, by APL1 policing of bacterial escape from the epithelial barrier. A humoral effect would be more consistent with the known route of APL1 protein secretion into the hemolymph in *A. gambiae* and *A. coluzzii* (Holm et al., 2012; Povelones et al., 2009; Mitri et al., 2015), although important differences in *A. stephensi* cannot be ruled out. Interestingly, a recent report found that enteric bacteria in *Aedes aegypti*, including *Cedecea neteri* relatives, can become established intracellularly within the midgut epithelium and influence immune signaling (Hegde et al., 2019). Quantification and identification of *A. stephensi* hemolymph bacteria in the presence and absence of APL1 would indicate whether the difference in midgut levels of the OTUs, as measured here, is reflected in OTU escape to the systemic compartment.

Third, if the *Klebsiella-Cedecea* clade of *Enterobacteriaceae* provokes elevated mortality in *A. stephensi* in the absence of APL1 as we previously reported (Mitri et al., 2019), what is the mechanism of pathogenicity, and the effects on host physiology? Molecular markers such as levels of reactive oxygen species and AMPs need to be measured, as well as the lethal dose of bacteria. As mentioned above, manipulative experiments with pure cultures would first require complete genome sequences of the putative taxonomic swarm comprising the *Klebsiella-Cedecea* clade to generate diagnostic assays for the clade taxa within the *Anopheles* enteric compartment.

The microbiome has been conceptualized as an “ecosystem on a host leash” (Foster et al., 2017), where microbes interact among themselves for competitive advantage, and host mechanisms evolve to manage and maintain an adaptive consortium of microbes. Overgrowth by some taxa relative to others may produce a dysgenic state with deleterious effects for the host, due to specific toxic effects of the overgrown taxa and/or effects from destabilization of the consortium (Honda and Littman, 2016; Foster et al., 2017). Causative effects of the dysbiotic microbiome upon the host are seen in a number of pathologies (Gilbert et al., 2016; Honda and Littman, 2016; Byndloss et al., 2017; Surana and Kasper, 2017), but there are fewer studies identifying specific host mechanisms associated with management of particular commensal microbes, as we report here. In one relevant case, the loss of the bacterial flagellin sensor TLR5 in mice provoked multiple pathologies associated with metabolic syndrome, and also shifted the abundance of a suite of about 100 bacterial taxa in the enteric microbiome (Vijay-Kumar et al., 2010). A study in *D. melanogaster* ablated the function of the gene Caudal, which normally protects the microbiome by repressing antimicrobial peptide gene expression in the digestive tube (Ryu et al., 2008). Depletion of Caudal caused widespread shifts within the microbiome community, including of rare taxa that became abundant and the reverse. However, the current results are distinct in that APL1 depletion in *A. stephensi* is associated with a focused rather than global effect upon enteric taxa, but much remains to be done to understand the biological impact for host physiology and mosquito transmission of *Plasmodium* and arboviruses.

## Materials and Methods

### Mosquitoes

*Anopheles stephensi* SDA-500 strain was initiated in Pakistan (Feldmann and Ponnudurai, 1989) and is maintained in the insectaries of the CEPIA platform at the Institut Pasteur, Paris. Mosquitoes were reared under standard conditions at 26°C and 80% relative humidity, with a 12 h light/dark cycle and continuous access to 10% sucrose solution in cotton pads (Mitri et al., 2015). *Anopheles* larvae used for sequencing of the V4 hypervariable region of the 16S ribosomal RNA gene were collected in Goundry, Burkina Faso (latitude 12.5166876, longitude –1.3921092) using described methods (Coulibaly et al., 2016). DNA purified from individual larvae was typed individually for species by molecular diagnostic assay (Fanello et al., 2002). Amplicons of the V4 hypervariable region of the 16S rRNA gene were generated and sequenced as described below in pools of larvae.

### Gene Silencing

Double-stranded RNA (dsRNA) specific for target genes was synthesized using the T7 Megascript kit (Ambion) as described (Mitri et al., 2009) using indicated primers (Supplementary Table S6). For each targeted gene, 500 ng of dsRNA (but not more than 207 nl volume, depending on concentration) was injected into the thorax of cold-anesthetized 1-day-old female mosquitoes using a Nanoject II Auto-Nanoliter Injector (Drummond Scientific). Mosquitoes were injected with dsRNA specific for the target gene, or with control dsRNA containing the irrelevant sequence of GFP. The efficiency of gene silencing was monitored 4 days after dsRNA injection in pools of eight mosquitoes as follows. After total RNA extraction, cDNA synthesis was performed using M-MLV reverse transcriptase (Invitrogen) with random hexamers. For each sample, 1 μg of total RNA was used in each of three independent cDNA synthesis reactions. Triplicates were pooled and used as template for qPCR analysis. Real-time PCR was performed using an ABI Prism 7900HT sequence detector (Applied Biosystems). Reactions were prepared in 20 μl volumes using SYBR Green PCR master mix (Applied Biosystems) and 900 nM primers with three serial dilutions of cDNA, each dilution assayed in triplicate. Primers used for verification of gene silencing are indicated (Supplementary Table S6). PCR conditions were 95°C for 10 min followed by 40 cycles of 95°C for 15 s, 55°C for 15 s, and 60°C for 45 s. mRNA level was normalized to *A. stephensi* ribosomal protein rpS7 mRNA in each sample, and each gene silencing condition was compared to the control treated with GFP dsRNA.

### Mosquito Treatments, Replicates, and 16S V4 Amplicon Sequencing

Age-matched female *A. stephensi* mosquitoes raised as a cohort from a single oviposition of the parent colony were treated using the conditions as described above with antibiotic cocktail or sugar alone beginning at adult emergence (0-day-old), with injection of dsAPL1 or dsGFP at 3-days-old. Treatments by sample group are indicated in Supplementary Table S1. Midguts from 20 females per treatment condition were dissected 3 days after beginning of antibiotic treatment, or 4 days after dsRNA injection. Three biological replicate experiments were performed. Each replicate was carried out using a mosquitoes raised from a single large oviposition of the parent colony, and different replicates used mosquitoes grown from eggs laid by non-overlapping generations of the parent colony.

The 20 dissected midguts per sample were pooled for DNA extraction. Total DNA was extracted using the PowerSoil DNA Isolation kit (MoBio Laboratories Inc.) following the supplied protocol. Midguts were transferred into the PowerBead tube and homogenized three times using an MP FastPrep-96 Instrument (MP Biomedicals) at 1600 rpm for 1 min each. Total DNA was eluted in 100 ul PowerSoil elution buffer and stored at –20°C until downstream processing.

The V4 hypervariable region of the 16S rRNA gene was amplified and sequenced from midgut DNA pools. A 290 bp amplicon from the V4 region was amplified using the primers Meta_V4_515F and Meta_V4_806R (Supplementary Table S6). Amplicons were sequenced on the Illumina HiSeq platform as 2 × 150 paired-end reads at the University of Minnesota Genomics Center (UMGC, genomics.umn.edu), generating an average of 1.9 M reads per sample.

### Bacterial Microbiome-Wide Differential Abundance Analysis

Read filtering by sequence quality, sequence clustering to generate OTUs, and taxonomic annotation were performed using VSEARCH (Rognes et al., 2016) as implemented in the SHAMAN workflow (http://shaman.pasteur.fr, Quereda et al., 2016). A 97% sequence identity threshold was used for sequence clustering, yielding 457 OTUs across all samples (clustered OTU sequences, Supplementary File S1), of which 279 were annotated in the SILVA rRNA taxonomic database release 128 (Quast et al., 2013), and were provisionally named on that basis. The annotated raw OTU count matrix is presented in Supplementary Table S2. All subsequent analysis was performed with DESeq2 (v1.4.5) (Love et al., 2014) as implemented in SHAMAN. Of 4,072,977 total OTU raw counts across all samples, only 25,347 (0.6% of total OTU raw counts) map to the 178 OTUs that were not annotated in the SILVA database. Most of the unannotated OTUs were removed by the DESeq2 default “independent filtering” function, which removes OTUs with counts too low to generate reliable significant differences between conditions (Bourgon et al., 2010).

The raw OTU count values were normalized across all samples using the default method in DESeq2 (median of ratios method, the estimateSizeFactors function). Size factors of total OTU count numbers were in the range 0.5–2.5. The normalized OTU count matrix is presented in Supplementary Table S3. The relationship between estimated dispersion and normalized mean count values was evaluated by visual inspection as recommended, which indicated that local regression generated a better fit than parametric regression (Supplementary Figure S11). Therefore, the local regression analysis argument of DESeq2 was used to estimate differences in OTU abundance between experimental treatment variables (however, the analysis was also re-run using parametric regression for comparison, see below). All other default DESeq2 arguments were used. A public repository of input files necessary to reproduce and reanalyze the data using DESeq2 in SHAMAN is available at figshare (doi: 10.6084/m9.figshare.7356452).

We defined a multi-factor model that included the effects: dsRNA treatment, antibiotic treatment, and biological replicates, as well as the interaction between dsRNA and antibiotic treatment. The model design formula is summarized as (dsRNA + antibiotic + replicate + dsRNA:antibiotic). The interaction term accounts for non-additive effects of dsRNA and antibiotic treatment, and the biological replicate variable was treated as a batch effect. The model tested for two contrast vectors, (i) APL1 depletion versus APL1 presence, without antibiotics; and (ii) antibiotic treatment versus no antibiotics, with APL1 depletion. The default Wald test in DESeq2 was used to query for departure from the null hypothesis that fold-change for a given bacterial family or OTU within the above contrasts is zero. Nominal *p*-values were adjusted by the Benjamini and Hochberg procedure (Benjamini and Hochberg, 1995). The *DESeq* function within DESeq2 generated a results table listing the mean of normalized counts across samples (baseMean counts), fold-change, log2 fold-change, and adjusted *p*-values for each bacterial family or OTU (Supplementary Table S4 for the contrast effect of APL1 depletion in the absence of antibiotics, and Supplementary Table S5 for the contrast effect of antibiotic treatment in the absence of APL1 function). Diagnostic PCoA plots were generated using Bray-Curtis dissimilarity, with statistical analysis by permutational analysis of variance (PERMANOVA).

For subsequent analysis, an abundance cut-off was made that distinguishes 8 major OTUs comprising 93% of total *A. stephensi* enteric bacterial abundance, defined as the mean of normalized counts across all samples (Supplementary Figure S2). The first minimum in the OTU density curve, at the inflection value of 1903 counts, was used as a natural gap to make this cut-off.

An alternative analysis modified only by use of the default argument, parametric regression (instead of local regression) generated consistent results. Comparing the two taxa highlighted in Results, OTU3_Klebsiella and OTU281_Cedecea, parametric regression values for APL1 depletion contrast for OTU281_Cedecea: fold-change 1.699, adjusted *p*-value 0.439566; OTU3_Klebsiella: fold-change 4.051, adjusted *p*-value 0.020939; antibiotic treatment contrast for OTU281_Cedecea: fold-change 8.221, adjusted *p*-value 7.29E-07; OTU3_Klebsiella: fold-change 9.403, adjusted *p*-value 2.86E-08.

### Quantitative PCR Assays for *Klebsiella* and *Cedecea* Taxa

Before dissection, mosquitoes were washed in 75% ethanol for 5 min to kill mosquitoes and to fix surface bacteria to the cuticle. Mosquitoes were then washed three times in sterile phosphate buffered saline to wash off non-attached bacteria. Midguts were dissected and frozen immediately on dry ice in two groups of 20 mosquitoes per condition and stored at −80°C until processing. DNA was extracted using the PowerSoil DNA Isolation kit (MoBio Laboratories Inc.). Primers OTU3_Klebsiella _ccgg411_F and OTU3_Klebsiella _ccgg585_R were used for quantification of OTU3_Klebsiella, and primers OTU281_Cedecea _ ttat460_F and OTU281_Cedecea _ttat638_R for quantification of OTU281_Cedecea. Primers 16S_V4q_F and 16S_V4q_R were used for quantification of total bacterial load. Primer sequences given in Supplementary Table S6.

qPCR was performed on a CFX96 qPCR Detection System (Bio-Rad) using the KAPA SYBR^®^ FAST qPCR Master Mix kit, following the manufacturer’s instructions. Each sample was run in triplicate. PCR conditions were: 95°C for 10 min followed by 40 cycles of 95°C for 15 s, 60°C for 1 min and 72°C for 30 s. The internal control was ribosomal protein S7 gene.

### Phylogenetic Placement of OTUs

For phylogenetic analysis, a reference 16S rRNA gene tree was reconstructed with 2807 full-length 16S rRNA gene sequences from complete prokaryotic genomes available at GenBank in March 2016. Sequences were aligned with Pynast (Caporaso et al., 2010) against a curated template 16S rRNA gene alignment from the Greengenes database (DeSantis et al., 2006). A Maximum-Likelihood phylogenetic tree was reconstructed with RAxML (Stamatakis, 2006) using the GTR model of sequence evolution and the Gamma model of rate heterogeneity between sites. Sequences of 16S V4 Illumina amplicons and the qPCR extended amplicons were aligned with the reference 16S rRNA gene alignment used to build the reference tree using Pynast. This extended alignment was used to place 16S V4 Illumina amplicons and the qPCR extended amplicons on the reference tree using the Evolutionary Placement Algorithm of RAxML, which sequentially places each short query sequence (read) at each edge of a reference tree previously constructed with longer sequences and calculates the likelihood of the resulting tree (Berger et al., 2011). The resulting trees were visualized with the R package Analyses of Phylogenetics and Evolution, APE (Paradis et al., 2004).

### Shotgun Metagenomic Sequence Analysis

To enrich for APL1-responsive OTUs, we combined midgut DNA from all above dsAPL1-treated samples and performed shotgun metagenomic sequencing. The shotgun sequencing library was constructed and sequenced at UMGC by Next-Seq (Illumina) in High Output mode with 2 × 150 PE reads. This generated 461,653,619 sequencing reads (more than 138 trillion base pairs of sequence). Shotgun metagenomic reads were quality-filtered with Trimmomatic version 0.35 (Bolger et al., 2014), set to trim sequences when the average quality per base dropped below 15 within a 4 bp sliding window, and removing reads smaller than 36 bp. Mosquito reads were removed prior to analysis by mapping quality-filtered reads against genome assemblies of *A. stephensi* strain SDA-500 (GCA_000349045.1) and *A. stephensi* strain Indian (GCA_000300775.2) with BWA-MEM version 0.7.15 (Li and Durbin, 2009). Non-mosquito reads were taxonomically binned with Centrifuge version 1.0.3 (Kim et al., 2016) searching against a reference database containing 8682 prokaryotic, viral and fungal reference genomes from NCBI GenBank. The identities and estimates of proportional abundance of taxa in the *Cedecea* and *Klebsiella* fractions generated by Centrifuge were visualized in the form of Krona diagrams (Ondov et al., 2011).

## Data Availability Statement

All sequence files are available from the EBI European Nucleotide Archive database (http://www.ebi.ac.uk/ena/) under ENA study accession number PRJEB30867. Assembled bacterial OTU sequences are available in this article, Supplementary File S1.

## Ethics Statement

The protocol for the ethical treatment of the animals used in this study was approved by the research animal ethics committee of the Institut Pasteur, “C2EA-89 CETEA Institut Pasteur” as protocol number B75-15-31. The Institut Pasteur ethics committee is authorized by the French Ministry of Higher Education and Research (MESR) under French law N° 2001-486, which is aligned with Directive 2010/63/EU of the European Commission on the protection of animals used for scientific purposes.

## Author Contributions

CM, EB, EBC, KE, WG, N’FS, MR, and KV designed the research. CM, EB, EBC, SV, AG, KE, IH, CD, EB-F, WG, N’FS, and MR performed the research. CM, EB, EBC, SV, AG, KE, IH, CD, WG, MR, and KV analyzed the data. CM, EB, EBC, SV, AG, KE, IH, N’FS, MR, and KV wrote the manuscript.

## Conflict of Interest

The authors declare that the research was conducted in the absence of any commercial or financial relationships that could be construed as a potential conflict of interest.

## Supplementary Material

FIGURE S1 Principal Coordinates Analysis of OTU distributions in three biological replicates. PCoA analysis of the samples subjected to microbiome sequencing for comparison of OTU distributions between biological replicates: replicate 1 (Rep1), replicate 2 (Rep2), replicate 3 (Rep3). Permutational multivariate analysis of variance (PERMANOVA) *p* = 0.019.

FIGURE S2 Distribution of mean of normalized counts for all OTUs across all experimental samples. **(A)** Frequency distribution of OTU abundance, indicated as the mean of normalized counts of all samples (baseMean counts). **(B)** MA-plot of OTU log2 fold-change, of APL1 depleted samples treated with dsAPL1 as compared to dsGFP treated controls, without antibiotics, x-axis as in panel **(A)**. Vertical dashed line in both panels indicates threshold distinguishing the 8 OTUs comprising 93% of total *A. stephensi* enteric bacterial abundance. The positions of OTU3_Klebsiella (orange arrow OTU3) and OTU281_Cedecea (blue arrow OTU281) are indicated.

FIGURE S3 APL1 sensitive candidate OTUs belong to a narrow clade of *Enterobacteriaceae*. For phylogenetic placement, consensus sequences of the OTU3_Klebsiella and OTU281_Cedecea 16S V4 Illumina amplicons were clustered in a reference 16S ribosomal gene phylogenetic tree of 2807 full-length 16S ribosomal RNA gene sequences from GenBank complete prokaryotic genomes. OTUs identified through metagenomic sequencing and qPCR amplicons were placed on a reference maximum likelihood tree of complete 16S rRNA genes with the Evolutionary Placement Algorithm implemented in RAxML. The tree on the left is colored to indicate bacterial genus (color key on lower right), and context is indicated by the black rectangle framing a portion of the Gammaproteobacteria branch, which is expanded in the tree to the right. Positions of 16S V4 Illumina amplicon sequences obtained for OTU3_Klebsiella and OTU281_Cedecea are highlighted in blue and indicated with blue arrows, and positions of qPCR amplicons corresponding to the same OTUs are highlighted in purple and indicated with purple arrows.

FIGURE S4 Design of discriminant qPCR primers external to original 16S V4 Illumina amplicons. OTU3_Klebsiella and OTU281_Cedecea 16S V4 Illumina amplicons were aligned with nearest relatives from the phylogenetic tree of full-length 16S ribosomal gene sequences shown in Supplementary Figure S3. Sequence information extending beyond the 16S V4 Illumina amplicons was used to design discriminant qPCR primers (termini of V4 amplicons indicated in the sequences labeled, OTU281_Cedecea V4 Amplicon, and OTU3_Klebsiella V4 Amplicon). The location of OTU281_Cedecea qPCR primers are boxed in black frame and the OTU3_Klebsiella qPCR primers boxed in red frame. Forward primers are displayed at the beginning of the alignment, and reverse primers toward the end. Primer sequences listed in Supplementary Table S6.

FIGURE S5 Correlation between quantitative PCR normalization using mosquito DNA or total bacterial abundance. Differential abundance of OTUs in *A. stephensi* after APL1 depletion was measured by quantitative PCR as the ratio of OTU abundance after APL1 depletion by dsAPL1 treatment as compared to control dsGFP treatment. Plot compares normalization using *A. stephensi* ribosomal protein rpS7 gene or total 16S rRNA gene DNA as internal calibrator. Normalization using either internal calibrator was concordant (*r*^2^ = 0.9991).

FIGURE S6 Bacterial species assignments of metagenomic shotgun sequence reads from *Anopheles stephensi* midguts. Composition of shotgun sequence reads from midguts of APL1-depleted *A. stephensi*. *Cedecea* and *Klebsiella* reads were taxonomically binned and searched against assembled bacterial genomes in the GenBank database to determine the species composition of *Cedecea* and *Klebsiella* midgut shotgun fractions. Krona plot of *Enterobacteriaceae* indicates the *Cedecea* and *Klebsiella* species present in the annotated GenBank genomes.

FIGURE S7 Alignment of 16S V4 Illumina amplicons, qPCR extended amplicons, and complete 16S genes from annotated GenBank assemblies of *Cedecea* species identified in the midgut shotgun dataset. The qPCR amplicon for OTU281_Cedecea is external to the OTU281_Cedecea 16S V4 Illumina amplicon for positions 1-79. The qPCR forward primer is external to the V4 Illumina amplicon, and the reverse primer includes nucleotide positions diagnostic for the TTAT oligotype, including the 3′ position of the primer. The qPCR primers display 100% identity to 16S genes corresponding to 5/5 available *C. neteri* genome assemblies in GenBank (100%), and 1/1 (100%) *Cedecea* NFIX57 genome assemblies, and thus the qPCR assay should efficiently detect these taxa. Boxes indicate four fixed nucleotide variants diagnostic for the *Cedecea* oligotype, TTAT. Sequence list: *Cedecea* rRNA gene sequences indicated with GenBank record identifier; sequence of qPCR extended amplicons indicated as OTU3_Klebsiella qPCR amplicon and OTU281_Cedecea qPCR amplicon; sequence of V4 Illumina amplicons indicated as OTU3_Klebsiella V4 amplicon and OTU281_Cedecea V4 amplicon; sequence of qPCR primers (Supplementary Table S6) indicated as Primer OTU281_Cedecea_F and Primer OTU281_Cedecea_R.

FIGURE S8 Alignment of 16S V4 Illumina amplicons, qPCR extended amplicons, and complete 16S genes from annotated GenBank assemblies of *Klebsiella* species identified in the midgut shotgun dataset. As in Supplementary Figure S7, but for *Klebsiella* species. The qPCR amplicon for OTU3_Klebsiella is external to the OTU3_Klebsiella 16S V4 Illumina amplicon for positions 1-96. The qPCR forward primer is external to the V4 Illumina amplicon. The qPCR primers display 100% identity and should efficiently amplify the 16S gene in:16/61 of *K. michiganensis* genomes (26.22%).

FIGURE S9 Alignment of 16S V4 Illumina amplicons, qPCR extended amplicons, and complete 16S genes from annotated GenBank assemblies of *Klebsiella* species identified in the midgut shotgun dataset. As in Supplementary Figure S7, but for *Klebsiella* species. The qPCR amplicon for OTU3_Klebsiella is external to the OTU3_Klebsiella 16S V4 Illumina amplicon for positions 1-96. The qPCR forward primer is external to the V4 Illumina amplicon. The qPCR primers display 100% identity and should efficiently amplify the 16S gene in:1/86 of *K. oxytoca* genomes (1.13%).

FIGURE S10 Alignment of 16S V4 Illumina amplicons, qPCR extended amplicons, and complete 16S genes from annotated GenBank assemblies of *Klebsiella* species identified in the midgut shotgun dataset. As in Supplementary Figure S7, but for *Klebsiella* species. The qPCR amplicon for OTU3_Klebsiella is external to the OTU3_Klebsiella 16S V4 Illumina amplicon for positions 1-96. The qPCR forward primer is external to the V4 Illumina amplicon. The qPCR primers display 100% identity and should efficiently amplify the 16S gene in:1/1 of *K.sp.* LTGPAF-6F genomes (100%). In each alignment, boxes indicate four fixed nucleotide variants diagnostic for the *Klebsiella* oligotype, CCGG. Sequences: *Klebsiella* rRNA gene sequences indicated with GenBank record identifier; sequence of qPCR extended amplicons indicated as OTU3_Klebsiella qPCR amplicon and OTU281_Cedecea qPCR amplicon; sequence of V4 Illumina amplicons indicated as OTU3_Klebsiella V4 amplicon and OTU281_Cedecea V4 amplicon; sequence of qPCR primers (Supplementary Table S6) indicated as Primer OTU3_Klebsiella_F and Primer OTU3_Klebsiella_R.

FIGURE S11 Analysis of OTU normalized mean count dispersion. **(A)** Local regression and **(B)** parametric regression analysis options were evaluated using DESeq2 for goodness of fit for dependence between normalized mean count values and estimated dispersion.

TABLE S1 Sample list for sequencing of V4 hypervariable region amplicon of the 16S ribosomal RNA gene.

TABLE S2 Raw mean counts for all experimental samples for all OTUs with matches in the SILVA database release 128.

TABLE S3 Normalized mean counts for all experimental samples for all OTUs with matches in the SILVA database release 128.

TABLE S4 Significance table for the comparison between APL1 depletion versus dsGFP treated controls without antibiotic, for bacterial families and OTUs.

TABLE S5 Significance table for the comparison between antibiotic treatment versus no antibiotic with APL1 depletion, for bacterial families and OTUs.

TABLE S6 Sequence of primers. Sequence of the primers (5′-3′) used for the synthesis of double-stranded RNA for gene silencing (name preceded by “T7”), and for verification of silencing efficiency by quantitative RT-PCR (name preceded by “Ast”). Sequences preceded by “16S” used for quantitative PCR of total 16S rRNA abundance in mosquito midgut DNA. Sequences preceded by “OTU” used for quantitative PCR of qPCR extended amplicons for each indicated OTU. Sequences preceded by “Meta” used for amplification of the V4 hypervariable region of the 16S rRNA gene, with the bacterial 16S rRNA gene-specific portion indicated in bold.

FILE S1 FASTA-format database file of 457 clustered OTU sequences from *Anopheles stephensi* midgut. The V4 hypervariable region of the 16S ribosomal RNA (rRNA) gene was amplified and sequenced from DNA of *A. stephensi* midguts dissected from mosquitoes treated as described. A total of 457 OTUs were identified among all samples and treatment conditions combined, using VSEARCH as implemented in the SHAMAN workflow with a clustering threshold of 97% sequence identity.
