# Supplementary material for: Leucine-Rich Immune Factor APL1 Is Associated With Specific Modulation of Enteric Microbiome Taxa in the Asian Malaria Mosquito Anopheles stephensi
Source: Front Microbiol. 2020 Feb 26;11:306. doi: 10.3389/fmicb.2020.00306 (PMC7054466; doi:10.3389/fmicb.2020.00306)
Supplement: TABLE S6 — Sequence of primers. Sequence of the primers (5′-3′) used for the synthesis of double-stranded RNA for gene silencing (name preceded by “T7”), and for verification of silencing efficiency by quantitative RT-PCR (name preceded by “Ast”). Sequences preceded by “16S” used for quantitative PCR of total 16S rRNA abundance in mosquito midgut DNA. Sequences preceded by “OTU” used for quantitative PCR of qPCR extended amplicons for each indicated OTU. Sequences preceded by “Meta” used for amplification of the V4 hypervariable region of the 16S rRNA gene, with the bacterial 16S rRNA gene-specific portion indicated in bold. [file Table_6.DOCX]

| T7_Ast_ APL1_F | TAATACGACTCACTATAGGatcaaggatccgatgattccg |
| --- | --- |
| T7_Ast_ APL1_R | TAATACGACTCACTATAGGatgttggattactttattagc |
| Ast_APL1q_F | caaaggcccagcatgagcag |
| Ast_APL1q_R | tgtgcggaatcatcggatcc |
| Ast_S7q_F | AGGCGATCATCATCTACGTGC |
| Ast_S7q_R | caatgaacacgacgtgctt |
| 16S_V4q_F | GTGCCAGCMGCCGCGGTAA |
| 16S_V4q_R | GGACTACHVGGGTWTCTAAT |
| OTU281_Cedecea _ ttat460_F | RTTAAGGTTAATAACCTTNG |
| OTU281_Cedecea _ttat638_R | CCCTCTACAAGACTCAAGCT |
| OTU3_Klebsiella _ccgg411_F | TTCAGCGGGGAGGAAGGTGT |
| OTU3_Klebsiella _ccgg585_R | ACCCCCCTCTACAAGACTC |
| Meta_V4_515F | TCGTCGGCAGCGTCAGATGTGTATAAGAGACAG**GTGCCAGCMGCCGCGGTAA** |
| Meta_V4_806R | GTCTCGTGGGCTCGGAGATGTGTATAAGAGACAG**GGACTACHVGGGTWTCTAAT** |

**S6 Table. Sequence of primers.** Sequence of the primers (5’-3’) used for the synthesis of double-stranded RNA for gene silencing (name preceded by “T7”), and for verification of silencing efficiency by quantitative RT-PCR (name preceded by “Ast”). Sequences preceded by “16S” used for quantitative PCR of total 16S rRNA abundance in mosquito midgut DNA. Sequences preceded by “OTU” used for quantitative PCR of qPCR extended amplicons for each indicated OTU. Sequences preceded by “Meta” used for amplification of the V4 hypervariable region of the 16S rRNA gene, with the bacterial 16S rRNA gene-specific portion indicated in bold.
